# Diversity of Cervical Microbiota in Asymptomatic *Chlamydia trachomatis* Genital Infection: A Pilot Study

**DOI:** 10.3389/fcimb.2017.00321

**Published:** 2017-07-14

**Authors:** Simone Filardo, Marisa Di Pietro, Maria G. Porpora, Nadia Recine, Alessio Farcomeni, Maria A. Latino, Rosa Sessa

**Affiliations:** ^1^Section of Microbiology, Department of Public Health and Infectious Diseases, University of Rome “Sapienza” Rome, Italy; ^2^Department of Gynecology, Obstetrics and Urology, University of Rome “Sapienza” Rome, Italy; ^3^Section of Statistics, Department of Public Health and Infectious Diseases, University of Rome “Sapienza” Rome, Italy; ^4^Unit of Bacteriology, STIs Diagnostic Centre, Sant'Anna Hospital Turin, Italy

**Keywords:** *Chlamydia trachomatis*, asymptomatic infection, cervical microbiota, next-generation sequencing, bacterial diversity

## Abstract

*Chlamydia trachomatis* genital infection continues to be an important public health problem worldwide due to its increasing incidence. *C. trachomatis* infection can lead to severe sequelae, such as pelvic inflammatory disease, obstructive infertility, and preterm birth. Recently, it has been suggested that the cervico-vaginal microbiota may be an important defense factor toward *C. trachomatis* infection as well as the development of chronic sequelae. Therefore, the investigation of microbial profiles associated to chlamydial infection is of the utmost importance. Here we present a pilot study aiming to characterize, through the metagenomic analysis of sequenced 16s rRNA gene amplicons, the cervical microbiota from reproductive age women positive to *C. trachomatis* infection. The main finding of our study showed a marked increase in bacterial diversity in asymptomatic *C. trachomatis* positive women as compared to healthy controls in terms of Shannon's diversity and Shannon's evenness (*P* = 0.031 and *P* = 0.026, respectively). More importantly, the cervical microbiota from *C. trachomatis* positive women and from healthy controls significantly separated into two clusters in the weighted UniFrac analysis (*P* = 0.0027), suggesting that differences between the two groups depended entirely on the relative abundance of bacterial taxa rather than on the types of bacterial taxa present. Furthermore, *C. trachomatis* positive women showed an overall decrease in *Lactobacillus* spp. and an increase in anaerobes. These findings are part of an ongoing larger epidemiological study that will evaluate the potential role of distinct bacterial communities of the cervical microbiota in *C. trachomatis* infection.

## Introduction

In recent years, a growing body of evidence highlighted that the cervico-vaginal microbiota plays a key role in the outcomes of genital infections (Ma et al., 2012). In healthy reproductive women, the cervico-vaginal microbiota is mostly populated by *Lactobacillus* spp., whose presence can be considered as a hallmark of healthy conditions (Ma et al., 2012; van de Wijgert et al., 2014). Indeed, to date, it is widely accepted that cervico-vaginal lactobacilli may have a significant impact on individual susceptibility to genital infections (Brotman, 2011; Buve et al., 2014). Specifically, lactobacilli are the main host defense factor within the cervico-vaginal ecosystem, since they are able to limit the growth of potential pathogens through different mechanisms, including the competitive exclusion, competition for nutrients, anti-microbial compound production, the immune system activation as well as the maintenance of a low vaginal pH (Mijac et al., 2006; Vielfort et al., 2008; O'Hanlon et al., 2013; Doerflinger et al., 2014).

In addition to lactobacilli, other microorganisms populate the cervico-vaginal microbiota, like, for example, *Prevotella* spp., *Gardnerella vaginalis*, and *Atopobium vaginae*. The replacement of the *Lactobacillus* spp., with these microorganisms may result in the switch from a healthy genital microbiota to a dysbiosis defined as bacterial vaginosis (BV) (Srinivasan et al., 2012; Dols et al., 2016). BV has been associated, in turn, with an increased risk of acquiring sexually transmitted diseases, including *Chlamydia trachomatis* (Hillier et al., 1992; Wiesenfeld et al., 2003; Brotman et al., 2010; Petrova et al., 2015). *C. trachomatis* is the leading cause of bacterial sexually transmitted diseases and it is responsible for cervicitis, salpingitis, and endometritis (World Health Organization, 2012). In particular, it has been observed that women with BV may be at a higher risk for *C. trachomatis* cervicitis, as suggested by a high Nugent score (>7) in ~85% of *C. trachomatis* infected women (Yoshimura et al., 2009). More interesting, there is the evidence that *C. trachomatis* genital infection increases the risk of acquiring and transmitting human immunodeficiency virus (HIV) by three- to four-fold (Galvin and Cohen, 2004; Buckner et al., 2016) as well as of developing human papilloma virus (HPV) induced cervical carcinoma (Jensen et al., 2014; Silva et al., 2014).

Nowadays, *C. trachomatis* infection continues to be an important public health problem worldwide because of its increasing incidence (World Health Organization, 2012). Indeed, a 24% rise in the number of new cases has been observed since 2008 and this is likely due to a higher awareness of the impact of *C. trachomatis* infection on female reproductive outcomes (Newman et al., 2015). In fact, *C. trachomatis* genital infection can lead to severe sequelae, such as pelvic inflammatory disease, ectopic pregnancy, obstructive infertility as well as miscarriages and preterm birth (Mylonas, 2012; Lanjouw et al., 2016), and the risk of these long-term reproductive sequelae is likely increased by the high rate (80%) of asymptomatic infections (Sessa et al., 2015; Lanjouw et al., 2016).

Recently, several studies have demonstrated the protective activity of vaginal *Lactobacillus* spp., as well as of other host defense factors present in the healthy cervico-vaginal micro-environment, toward *C. trachomatis* infection (Mastromarino et al., 2014; Nardini et al., 2016; Sessa et al., 2017a,b). Specifically, it has been showed that *Lactobacillus crispatus* and *Lactobacillus brevis* are able to interfere with the early phases of *C. trachomatis* infection as well as to inhibit its intracellular replication, preventing the dissemination of the infection in the host (Mastromarino et al., 2014; Sessa et al., 2017a). Therefore, the complex interplay between the cervico-vaginal microbiota and *C. trachomatis* seems to be critical for the onset of chlamydial infection and the development of severe sequelae.

Given that alterations in microbial communities of the genital tract may affect the susceptibility to *C. trachomatis*, investigating the composition of cervical microbiota associated to chlamydial infection is of the utmost importance. Therefore, here we present a pilot study aiming to characterize, through the metagenomic analysis of sequenced 16s rRNA gene amplicons, the cervical microbiota from reproductive age women with asymptomatic *C. trachomatis* infection.

## Materials and methods

### Study design and sample collection

From May to September 2016, 45 consecutive women of Italian origin, attending the Department of Gynaecology, Obstetrics and Urology at “Sapienza” University of Rome for regular check-ups, were enrolled in this study.

All women were not pregnant, of reproductive age, and regularly menstruating. Women with recent or current antibiotic and/or hormonal medications (oral or topic), as well as use of probiotics and/or prebiotics, were excluded from the study.

All study participants gave their written informed consent prior to sampling and provided a detailed personal, medical and gynecological history. This study design and protocol was approved by the Umberto I University Hospital ethical committee (reference number 367/16) and was conducted according to the principles expressed in the Declaration of Helsinki.

From each woman, one endo-cervical swab (FLOQ swabs, Copan) was collected for *C. trachomatis* testing as previously described (Frieden et al., 2015), and for the metagenomic analysis. All the women negative to *C. trachomatis* infection were also screened for *Neisseria gonorrhoeae, Trichomonas vaginalis*, Mycoplasma, Candida, HPV, and HSV-2 infections as previously described (Frieden et al., 2015), in order to include exclusively women with healthy genital conditions.

All study participants were also examined for BV and for the presence of symptoms. BV was assessed using Amsel criteria and confirmed using Gram stain criteria (Nugent score). Lastly, no women had any specific genital symptoms related to chlamydial infections.

All endo-cervical samples were placed in cryovials containing 1 mL of phosphate-buffered saline (PBS) and immediately stored at −80°C until further processing.

### Next-generation sequencing

Cervical samples were shipped in dry ice to Microsynth AG (Balgach, Switzerland) for DNA isolation, 16s rRNA gene amplification, Illumina MiSeq sequencing and initial bioinformatics analysis, consisting of raw data processing and taxonomic classification.

### DNA isolation

Cervical samples were thawed and transferred into 2 mL screwcap tubes (SarstedtInc, Germany) containing 10 μL of 4% Tween 80 (Downey et al., 2012). Tubes were, then, vortexed for 2 min on a TurboMix System (Scientific Industries, USA) and 350 μL of the sample were used for DNA isolation. Total DNA was extracted using the QIAmp Investigator kit (QIAGEN, USA), according to Manufacturer's Instructions. DNA was quantified by fluorescence spectroscopy (Quant-iT™ PicoGreen® dsDNA Assay Kit, Thermo Fisher, USA) and its integrity checked by agarose gel electrophoresis.

### 16s rRNA gene amplification and illumine MiSeq sequencing

Dual-indexed universal primers 341F (CCTACGGGNGGCWGCAG) and 802R (GACTACHVGGGTATCTAATCC; Illumina, USA) were utilized for the two-steps PCR amplification of the V3–V4 hypervariable regions of the 16s rRNA gene (16S/ITS Nextera two-step PCR kit, Illumina Inc., USA), according to Manufacturer's Instruction. Briefly, the first-step PCR was carried on to amplify the V3–V4 region of the 16s rRNA gene. The resulting PCR amplicons were then used for the second-step PCR for further amplification and inclusion of indexes (barcodes) as well as the Illumina sequencing adaptors. Cycling conditions were initial denaturation at 95°C for 3 min, followed by 20 cycles (for the first-step PCR) or 15 cycles (for the second-step PCR) of denaturation at 98°C for 20 s, annealing at 56°C for 30 s and elongation at 72°C for 30 s, one cycle of final elongation at 72°C for 5 min and a final cooling step to 10°C. The resulting PCR products were quantified by fluorescence spectroscopy (Quant-iT®PicoGreen® dsDNA Assay Kit, Thermo Fisher, USA), pooled in equimolar amounts and, then, purified with Agencourt AMpure-XP magnetic beads (Beckman Coulter, USA). The final library containing all the pooled samples was sequenced with version 2 of MiSeq Reagent Kit, 2 × 250 bp output, on a MiSeq desktop sequencer (Illumina, USA). One negative control (350 μL of sterile PBS) was included and subjected to the same procedures as the samples.

### Sequencing data analysis

MiSeq paired-end reads were subjected to demultiplexing and trimming of Illumina adaptor residuals using Illumina recommended parameter settings (Illumina MiSeq Reporter software, version 2.5.1.3). Sequences were aligned and joined using Fast Length Adjustment of SHort reads (FLASH, version 1.2.11) (Magoč and Salzberg, 2011) and primers trimmed off the aligned sequences using cutadapt (version 1.8.1) (Martin, 2011). Chimeric sequences were identified and removed by UCHIME (version 4.2) (Edgar et al., 2011) and only joined reads with an average quality score of 25 or higher were used for downstream analysis. Open reference operational taxonomic unit (OTU) clustering and taxonomic assignment were performed in QIIME (version 1.9.1) (Caporaso et al., 2010) using UCLUST against the SILVA rRNA reference database (version 111) (Edgar, 2010; Quast et al., 2013). An OTU was defined as a group of sequences with a similarity of 97% or more. OTUs with only one sequence (singletons) and those not found more than 10 times in any sample were excluded from the downstream analysis to minimize artifacts. OTUs that could not be identified to a species level using the reference database, were searched using BLAST and assigned to the deepest taxonomical level based on available published data.

Taxa summaries were performed in QIIME and all samples were normalized to the sample with the lowest read count for alpha and beta diversity comparisons. Shannon's diversity index and Shannon's evenness were used as metrics for alpha rarefaction analysis, which was performed in QIIME. Jackknifed principal coordinates analyses (PCoA) was used so to assure that our rarefaction selection was not the cause of the observed clustering patterns. PCoA analysis was based on unweighted and weighted UniFrac distance matrixes and computed in QIIME (Lozupone and Knight, 2005; Lozupone et al., 2007). For taxa comparisons, relative abundances based on all obtained reads were used.

Raw sequences were deposited into the NCBI's Sequence Read Archive (SRA) (https://trace.ncbi.nlm.nih.gov/Traces/sra/sra.cgi?), accession number SRP098954.

### Statistical analysis

Non-parametric *T*-test based on Monte Carlo permutations was used for alpha diversity comparisons, Kruskal–Wallis test for taxa level comparisons, and Adonis for category comparisons of distance matrixes, all calculated in QIIME. Benjamin–Hochberg false discovery rate (FDR) correction was used to correct for multiple hypothesis testing when necessary.

All remaining statistical calculations were performed in Excel (Microsoft, USA) and R 3.1.2 (R development core team). Chi-squared test was used for assessment of association of frequencies among groups (Fisher's exact test was used when any cell had expected values <5). Mann–Whitney *U*-test for non-parametric data was used for comparison of means. The single or multiple inference significance level was set to 5%.

## Results

### Study subject characteristics

Forty-five asymptomatic women of reproductive age were enrolled in this study. Ten women were positive to *C. trachomatis* (chlamydia group) and 35 were negative. Amongst the latter, 15 women were positive to other sexually transmitted infections (STIs) and, hence, were excluded from further analysis, whereas the remaining 20 were defined as healthy controls.

All 30 samples underwent 16s rRNA amplicon-based microbiome analysis and 16 samples were excluded from downstream analysis due to a number of reads <100.

Overall, the cervical microbiome analysis was performed on 14 cervical samples from 7 *C. trachomatis* positive women and 7 healthy controls. Population characteristics are summarized in Table 1. No statistically significant differences were observed between the two groups according to the risk factors commonly associated with *C. trachomatis* infection.

**Table 1:** Characteristics of the study population.

	**Healthy controls (*n* = 7)**	***C. trachomatis* positive women (*n* = 7)**	*P*-value^*^
Age (mean ±*SD*)	34.7 ± 6.8	26.9 ± 4.8	0.08
Smoking	28.6% (*n* = 2)	28.6% (*n* = 2)	1
First intercourse before age 16 years	14.3% (*n* = 1)	57.1% (*n* = 4)	0.26
New partner in the last 6 months	14.3% (*n* = 1)	71.4% (*n* = 5)	0.1
Multiple partners	28.6% (*n* = 2)	71.4% (*n* = 5)	0.29
Partner with past STI in the last 6 months	0%	14.3% (*n* = 1)	1
Bacterial vaginosis (Nugent score 7–10)	0%	42.9% (*n* = 3)	0.19
Past STI	0%	42.9% (*n* = 3)	0.19
Vaginal pH (mean ± *SD*)	4.78 ± 0.76	4.93 ± 0.95	0.8

*SD, standard deviation; STI, sexually transmitted infection*.

* *P-values were calculated using the Chi-squared test (Fisher's exact test when expected values <5) for the assessment of association of frequency among groups and the Mann–Whiney U-test for comparison of means*.

### Cervical microbiota composition

An average of 121,824 [median (IQR) 135,943 (77,506)] and 183,689 [median (IQR) 192,095 (17,689)] paired-end Illumina reads were analyzed per sample in healthy controls and chlamydia group, respectively. After the removal of singletons and rare OTUs, a total number of 22 OTUs from 16 genera were identified [median (IQR) = 9 (5) and 15 (5.5) for healthy controls and chlamydia groups, respectively]. The lowest read count was 29,613 and, hence, OTUs were randomly sub-sampled to 29,613 reads for further analysis to avoid sequencing bias.

**Figure 1:**
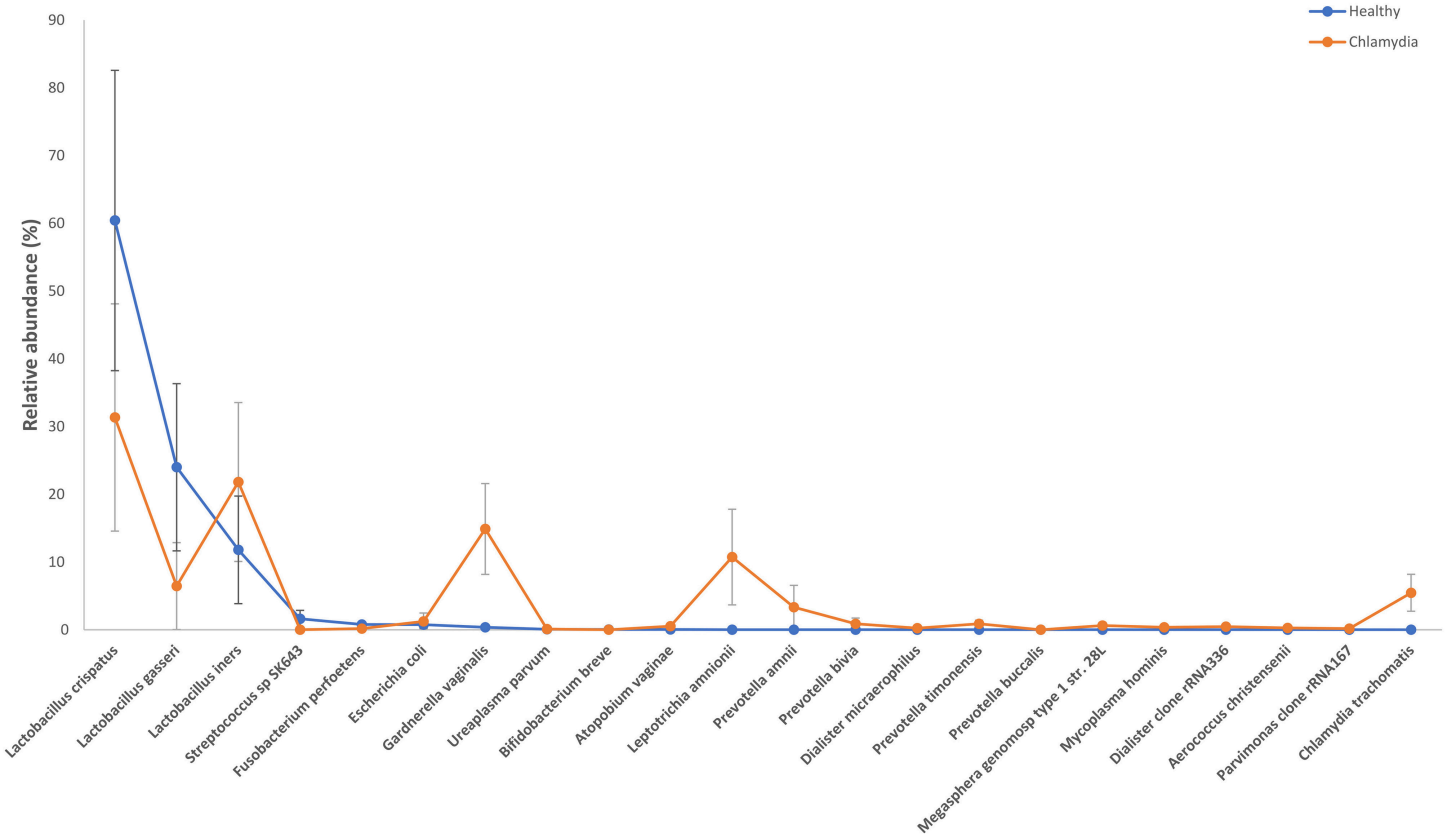
Relative abundance of the observed species in cervical samples from *C. trachomatis* positive women and healthy controls. All values are mean ± standard deviation. Kruskal–Wallis test with Benjamin–Hochberg false discovery rate (FDR) correction was used for taxa level comparisons between chlamydia-positive women and healthy control.

**Figure 2:**
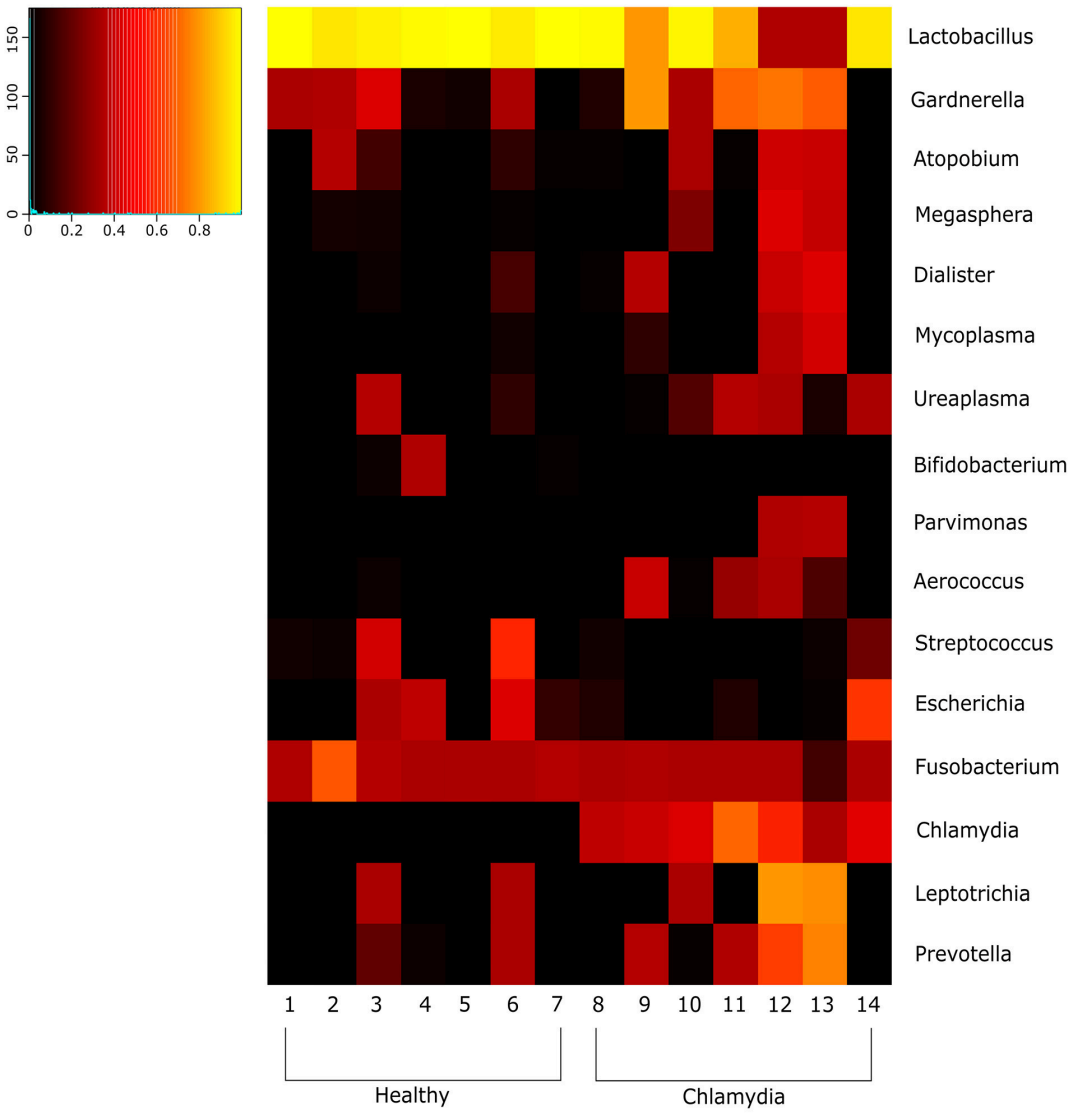
Heatmap indicating the changes at the genus level of the cervical microbial composition in *C. trachomatis* positive women and healthy controls. The legend below the heatmap represents each participant. The relative abundance of bacteria in each genus is indicated by a color gradient from black (low abundance) to yellow (high abundance).

The cervical microbiome in healthy controls was dominated by the phylum Firmicutes (median relative abundance 97.9%), while bacteria from the phyla Actinobacteria, Fusobacteria, Proteobacteria, and Tenericutes each accounted for <1% to total bacteria. On genus level, Lactobacillus was the single most abundant genus (median relative abundance 96.2%); other genera (*Gardnerella, Atopobium, Bifidobacterium*, etc.) were present, each contributing for <2% to total bacteria. Specifically, *L. crispatus* or *L. gasseri* were the predominant species in the majority of healthy women (86%), whereas in the remaining women *Lactobacillus iners* was the predominant species (Figures 1, 2).

Cervical samples from *C. trachomatis* positive women showed a decrease in the phylum Firmicutes (median relative abundance 60.2% as compared to 97.9% in healthy controls) and an increase in the phyla Actinobacteria (median relative abundance 16.1% as compared to 0.09% in healthy controls).

Amongst the Firmicutes, *Lactobacillus* spp. was less abundant in chlamydia groups than in healthy controls (median relative abundance 60 and 98%, respectively). In particular, *L. crispatus* or *L. gasseri* proportion was reduced in *C. trachomatis* positive women as compared to healthy controls (overall median relative abundance 89 and 32%, respectively; Figures 1, 2).

Actinobacteria were mostly represented by *G. vaginalis* (median relative abundance 14.3% as compared to 0.07% in healthy controls). *Prevotella* spp., belonging to the phylum Bacteroidetes, was increased as well, with a median relative abundance of 0.5 and 0.0001% in chlamydia group and healthy controls, respectively.

Lastly, the proportion of strict and/or facultative anaerobes was much higher in *C. trachomatis* positive women (median relative abundance 39.8%) as compared to healthy controls (median relative abundance 0.6%).

However, on phylum, class, order, family, genus, and OTU levels, no statistically significant differences were observed between chlamydia-positive patients and healthy controls.

### Alpha and beta diversity analyses

**Figure 3:**
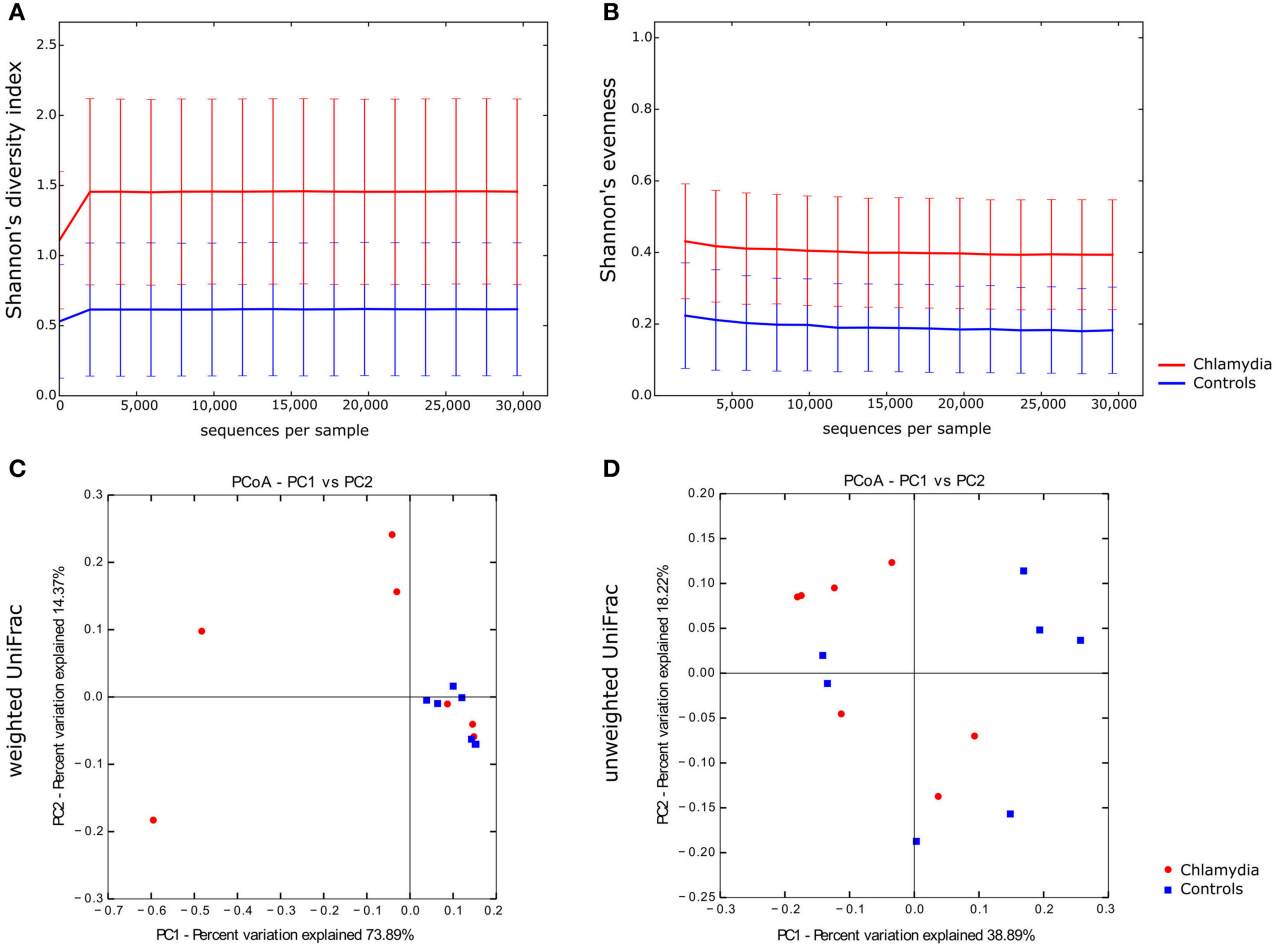
Alpha and beta diversity of the cervical microbiota from *C. trachomatis* positive women and healthy controls. Alpha rarefaction curves of Shannon's diversity index **(A)** and Shannon's evenness **(B)**. Samples were rarefied to the smallest observed number of reads (29,613). Principal coordinate analysis (PCoA) plots of weighted **(C)** and unweighted **(D)** UniFrac distances. Each dot represents the cervical bacterial community composition of one chlamydia-positive woman or healthy control. Groups were compared using Monte-Carlo permutations for alpha diversity and Adonis for beta diversity.

Alpha diversity analysis showed that *C. trachomatis* genital infection was significantly associated to an increased diversity of the cervical microbiota, as evidenced by Shannon's diversity index and Shannon's evenness (Figures 3A,B, *P* = 0.031 and *P* = 0.026, respectively).

In beta diversity analysis, cervical samples from healthy controls showed a tendency toward a statistically significant clustering, as compared to *C. trachomatis* positive women, in weighted UniFrac analysis (*P* = 0.0027), suggesting shifts in relative taxa abundance. In addition, the phylogenetic distances between *C. trachomatis* positive samples were significantly higher than the distances measured between healthy controls (Figure 3C, *P* = 0.000004). On the contrary, no clustering was observed when comparing unweighted UniFrac distance matrixes (Figure 3D, *P* > 0.05).

## Discussion

This study investigates the cervical microbiota composition of reproductive age women with asymptomatic *C. trachomatis* genital infection. Via the metagenomic analysis of 16s rRNA gene amplicons, we were able to determine the diversity and richness of cervical microbiota from *C. trachomatis* positive patients in comparison to women with a healthy cervical micro-environment. A variety of statistical approaches was used to provide a comprehensive evaluation of the cervical microbiota in both groups.

Our main findings highlighted a notable difference of the cervical microbiota of *C. trachomatis* positive women as compared to healthy controls. In particular, we showed that the cervical microbiota in asymptomatic *C. trachomatis* infection had a higher microbial diversity than the cervical microbiota in healthy controls, as evidenced by the marked increase in Shannon's diversity index as well as in Shannon's evenness (*P* = 0.031 and *P* = 0.026, respectively).

More importantly, we observed that the cervical microbiota from *C. trachomatis* positive women and from healthy controls significantly separated into two clusters in the weighted UniFrac analysis (*P* = 0.0027). Weighted UniFrac is a beta diversity measure that considers the phylogenetic distances as well as differences in the relative abundance of taxa between two microbial communities (Lozupone et al., 2007). On the contrary, no clustering was observed between the two groups according to the unweighted UniFrac analysis (*P* > 0.05), a qualitative distance metric that only detects differences in the presence or absence of lineages of bacteria (Lozupone and Knight, 2005). These findings suggest that differences between *C. trachomatis* positive women and healthy controls depended entirely on the relative abundance of the phylogenetic lineages rather than the types of lineages present. In fact, the taxa composition in both groups was comparable, as evidenced by the presence of the same genera (*Lactobacillus, Prevotella, Gardnerella*, etc.), whereas the relative abundance of each taxa greatly differed between the two groups.

As expected, we found that the cervical microbiota in healthy controls was dominated mainly by *Lactobacillus* species; specifically, *L. crispatus* and *L. gasseri* were found more frequently than *L. iners*.

Significant evidence suggest that the prevalence of specific *lactobacillus* species is strongly correlated with genital health (Ma et al., 2012; van de Wijgert et al., 2014; Petrova et al., 2015; Lewis et al., 2017). In fact, *L. crispatus*, as well as *L. gasseri*, is known to produce D-lactic acid, hydrogen peroxide (H_2_O_2_), bacteriocins and other anti-microbial compounds that help protecting against genital pathogens (Mitra et al., 2016).

*L. iners* as well is frequently found in healthy cervico-vaginal microbiota, although its beneficial role is still debated. Indeed, *L. iners* does not produce D-lactic acid as well as H_2_O_2_, and it has been detected in cervico-vaginal dysbiosis, suggesting that it may have a lower protective ability against pathogens as compared to other *lactobacillus* species (Mitra et al., 2016; Petrova et al., 2017).

In our study, unlike the healthy controls, *C. trachomatis* infection seemed to be accompanied by a decrease in the abundance of lactobacilli, and an increase in facultative and/or strict anaerobes, mostly *G. vaginalis, A. vaginae, P. amnii, P. timonensis*, and *Leptotrichia amnionii*. A similar alteration in cervico-vaginal microbiota related to *C. trachomatis* infection was also found by Ma et al. (2013) and van der Veer et al. (2017).

The shift from a microbiota dominated by *Lactobacillus* species to a microbiota characterized by the increased abundance of anaerobes is a condition known as BV (Srinivasan et al., 2012; Dols et al., 2016). Of note, in our study, 43% of *C. trachomatis* positive women was affected by BV and 67% of them had complex cervical microbial communities, characterized by a greater species diversity and a very low abundance of *Lactobacillus* species (<1%).

Concerning the structure of the microbial communities, it emerged, from the weighted UniFrac analysis, a higher inter-individual variability in *C. trachomatis*-positive women as compared to healthy controls, suggesting that a unique cervical microbial profile associated to *C. trachomatis* genital infection could not be determined. In fact, different cervical microbial profiles were observed; for example, in some cases, the cervical microbiota was constituted exclusively by anaerobes like *P. amnii, P. timonensis, G. vaginalis*, and *L. amnionii*, whereas, in other cases, by *L. gasseri, L. crispatus*, or *L. iners* plus a mixture of facultative and/or strict anaerobes.

*L. iners* was present in a higher number in *C. trachomatis* positive women than in healthy controls and this finding is in accordance with previous studies, showing that *C. trachomatis* positive women were more likely to have a microbiota dominated by *L. iners* (Ma et al., 2013; van der Veer et al., 2017). This may be explained by the ability of *L. iners* to survive in a wide range of pH and other metabolic stress-related conditions, adapting to an altered cervico-vaginal environment (Petrova et al., 2017).

The main strength of our study is the inclusion, as control group, exclusively of women with a healthy genital condition. Women positive to other genital infections were excluded, since pathogens, such as HPV, HIV, and *N. gonorrhoeae*, are known to be associated to an altered cervico-vaginal microbiota (Wiesenfeld et al., 2003; Petrova et al., 2013; Shannon et al., 2017). In addition, we excluded women with recent or current use of hormonal contraception and/or probiotic or prebiotic, all factors known to influence the cervico-vaginal microbial composition over-time (Brotman et al., 2014; Recine et al., 2016).

The application of strict inclusion criteria allowed us to greatly diminish the impact of the confounding bias associated to the selection of the study population, leading, unfortunately, to a small sample size. This is the main weakness of our study, that prevented us to define a unique cervical microbial profile associated to *C. trachomatis* infection. Notwithstanding, our data may add up valuable information to the ongoing research on the cervical microbiota associated to *C. trachomatis* genital infection, since these are the first data on the diversity and composition of the chlamydia-related endo-cervical microbiota.

In conclusion, our pilot study shows that *C. trachomatis*-positive women have a heterogeneous cervical bacterial community with increased species richness and diversity dominated by facultative and/or strict anaerobes. A larger epidemiological study investigating the richness and diversity of the cervical microbial composition related to specific *C. trachomatis* serotypes as well as chlamydial load is ongoing.

Our findings could have important clinical implications since they may be considered as biomarker for disease progression as well as for the restoring of a healthy cervical microbiota, so to reduce the pathological outcomes of *C. trachomatis* infection even if additional studies are needed.

## Author contributions

SF and MDP contributed equally to the manuscript. RS and MDP conceived and designed the study; MP, NR, and ML collected the samples and patients data; AF, SF analyzed the data; RS, MDP, and SF interpreted the results; RS, MDP, and SF wrote the manuscript. All authors reviewed and approved the manuscript.

### Conflict of interest statement

The authors declare that the research was conducted in the absence of any commercial or financial relationships that could be construed as a potential conflict of interest.
